# Sensitive Transfer-Free Wafer-Scale Graphene Microphones

**DOI:** 10.1021/acsami.2c03305

**Published:** 2022-04-27

**Authors:** Roberto Pezone, Gabriele Baglioni, Pasqualina M. Sarro, Peter G. Steeneken, Sten Vollebregt

**Affiliations:** † Laboratory of Electronic Components, Technology and Materials (ECTM), Department of Microelectronics, Delft University of Technology, 2628 CD Delft, The Netherlands; ‡ Kavli Institue of Nanoscience, Department of Quantum Nanoscience, Delft University of Technology, 2628 CD Delft, The Netherlands; § Department of Precision and Microsystems Engineering (PME), Delft University of Technology, 2628 CD Delft, The Netherlands

**Keywords:** graphene, microphone, membrane, MEMS, transfer free, wafer scale, high volume production

## Abstract

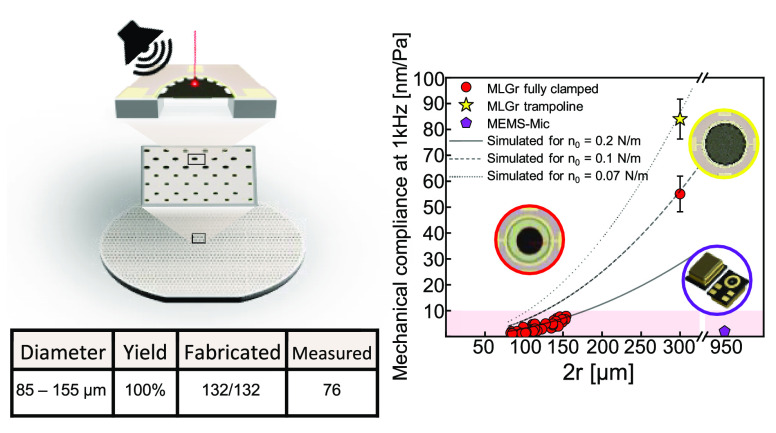

During the past decades micro-electromechanical microphones have largely taken over the market for portable devices, being produced in volumes of billions yearly. Because performance of current devices is near the physical limits, further miniaturization and improvement of microphones for mobile devices poses a major challenge that requires breakthrough device concepts, geometries, and materials. Graphene is an attractive material for enabling these breakthroughs due to its flexibility, strength, nanometer thinness, and high electrical conductivity. Here, we demonstrate that transfer-free 7 nm thick multilayer graphene (MLGr) membranes with diameters ranging from 85–155 to 300 μm can be used to detect sound and show a mechanical compliance up to 92 nm Pa^–1^, thus outperforming commercially available MEMS microphones of 950 μm with compliances around 3 nm Pa^–1^. The feasibility of realizing larger membranes with diameters of 300 μm and even higher compliances is shown, although these have lower yields. We present a process for locally growing graphene on a silicon wafer and realizing suspended membranes of patterned graphene across through-silicon holes by bulk micromachining and sacrificial layer etching, such that no transfer is required. This transfer-free method results in a 100% yield for membranes with diameters up to 155 μm on 132 fabricated drums. The device-to-device variations in the mechanical compliance in the audible range (20–20000 Hz) are significantly smaller than those in transferred membranes. With this work, we demonstrate a transfer-free method for realizing wafer-scale multilayer graphene membranes that is compatible with high-volume manufacturing. Thus, limitations of transfer-based methods for graphene microphone fabrication such as polymer contamination, crack formation, wrinkling, folding, delamination, and low-tension reproducibility are largely circumvented, setting a significant step on the route toward high-volume production of graphene microphones.

## Introduction

In the past 10 years, suspended graphene has attracted the interest of scientific and engineering communities due to the possibility of using its unique properties for sensor devices with novel functionality and improved performance like microphones, pressure, gas and Hall sensors.^1^ Low mass, a large surface-to-volume ratio, high electrical conductivity, a high Young’s modulus of 1 TPa, and a tensile strength up to 130 GPa are optimal for high performance micro- and nano-electromechanical system (MEMS/NEMS) technologies.^1^ Especially for microphones and pressure sensors, large membranes are needed to increase the sensitivity.^2,3^ Several works have explored the potential of graphene for microphone applications. Todorović et al. demonstrated a membrane with a 5 mm membrane diameter composed of 300 layers of chemical vapor deposition (CVD) graphene with a 10 dB higher sensitivity than commercial nickel-based microphones.^4^ Woo et al. realized a high-sensitivity microphone for hearing aids with a heterostructure membrane composed of 0.6 μm of graphene and 3 μm of PMMA on a 2.65 mm diameter membrane.^5^ Wittmann et al. reached almost similar performance as commercial silicon-based capacitive microphones using 15× smaller membranes (diameter 2*r* = 40 μm) made by only few-layer graphene.^6^ The high performance is mainly due to graphene’s high flexibility, low tension, and low out-of-plane stiffness, which results in large displacements in response to sound pressure. Significant effort has been made to improve the process to realize free-standing graphene by transferring the material to the target substrate by a carrier polymer with wet, dry, or semidry methods.^7^ The introduction of the inverted floating method (IFM) by Lee et al. and Akbari et al. helped fabricate large free-standing CVD graphene up to 2*r* = 500–750 μm.^8,9^ A combination of hydrogen bubbling transfer with thermal annealing by Chen et al.^10^ resulted in a suspended five-layer graphene membrane with a diameter of 1.5 mm. Carvalho et al. developed an anthracene sublimation assisted process achieving ten layers of CVD graphene suspended over 4 mm openings.^11^ Recently, six-layer graphene and 450 nm of PMMA were suspended over a closed cavity by HF vapor release (VHF) of the sacrificial layer after the graphene transfer.^12^ The dry release avoids liquids that usually introduce capillary forces that pull down the suspended part introducing ruptures or breaks.^13^ Moreover, exposing the graphene to wet HF might also lead to unwanted delamination of the graphene from the substrate.^14^ However, despite the very high aspect ratios and high crystallinity of the demonstrated free-standing membranes, the mentioned works do not provide a clear route toward industrialization of the devices because the transfer-based methods employed are not easily scalable toward high-volume wafer-level fabrication since no commercial equipment for transferring suspended graphene is available, and current methods often suffer from low yield, polymer contamination, cracks, and folding, leading to adhesion issues, especially for nonplanarized target substrates.^7^ This work proposes an alternative approach to overcome part of the previous limitations with a wafer-scale transferless method where multilayer graphene (MLGr) drums are grown and released on the same substrate. The method is potentially less prone to contamination and degradation of the membranes and less sensitive to topography, allowing it to be applied on nonplanarized surfaces. The realized multilayer graphene drums made by using this approach reach a peak mechanical compliance of ≈92 nm Pa^–1^ for 300 μm membranes with a yield of 18% and of ≈9 nm Pa^–1^ with a 100% yield of functional devices with diameters ranging between 85 and 155 μm. A study of the relation between the membrane diameter (2*r* = 85–300 μm) and the mechanical compliance, for more than 50 drums, demonstrates a good tension uniformity since it follows the expected quadratic dependency.^15^

## Experimental Section

### Design Concept and Material Compatibility

**Figure 1:**
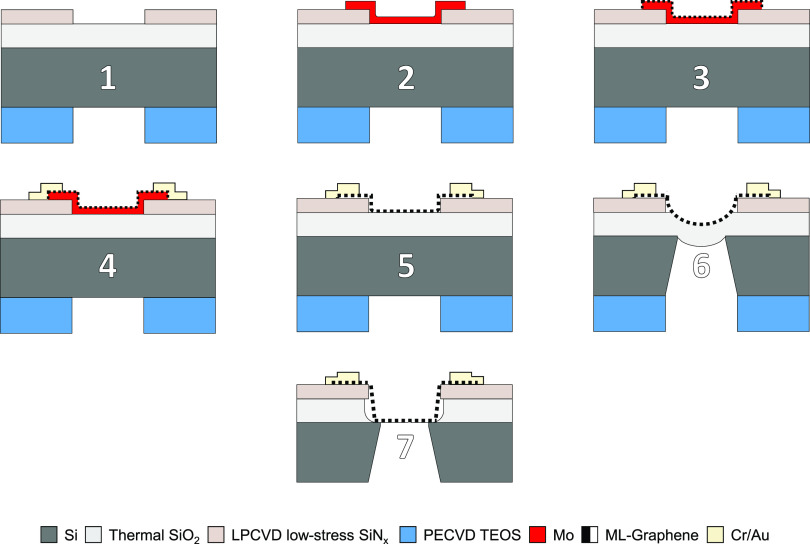
Main fabrication steps. (1) Patterning of the 110 nm LPCVD SiN_*x*_ layer (topside) and 5 μm PECVD TEOS layer (backside) by dry-etching. (2) Sputtered and dry-etch patterned 50 nm Mo layer. (3) Graphene synthesis at 935 °C at low pressure of 25 mbar. (4) Evaporated 20/200 nm Cr/Au layer after lift-off patterning. (5) Structure after sacrifical Mo wet-etch in H_2_O_2_. (6) Backside deep reactive ion etch (DRIE) of the silicon substrate. (7) Vapor HF etching of the SiO_2_ finally resulting in suspended MLGr membranes.

The device process design utilizes multilayer (ML) transfer-free graphene and vapor HF compatible materials to ensure the structures survive the sacrificial layer etch. Prepatterned Mo is used as a catalyst seed layer to locally synthesize the graphene on Si substrates along the same procedure described in earlier studies.^16,17^ ML-graphene (MLGr) is preferred here due to its higher mechanical strength that is required to realize membranes of sufficiently large diameter for microphone applications. Because the synthesis of the graphene is performed at 935 °C, all the materials are selected to have a sufficiently high melting point and mechanical stability during graphene CVD. Thermal SiO_2_ and LPCVD silicon-rich low-stress SiN_*x*_ are stable at these high temperature and show an etch selectivity of 40:1 to the vapor HF based on preliminary results and previous works on Si MEMS.^18,19^ The SiN_*x*_ layer is used as clamping support layer all around the edge of the graphene membrane and is also used to protect certain parts of the SiO_2_ from being etched during the silicon backside deep reactive ion etch (DRIE) and vapor HF sacrificial layer SiO_2_ release etch (Figure 1).

### Drum Fabrication

A layer of 110 nm LPCVD silicon-rich low-stress (SiH_2_Cl_2_ 315 sccm/NH_3_ 85 sccm) is deposited at 850 °C on top of 1 μm thermal oxide on a 100 mm silicon p-type wafer. The SiN_*x*_ is dry-etched in defined regions on the topside and entirely removed on the backside where a PECVD TEOS-based 5 μm is then deposited to work as etching mask for the silicon DRIE as shown Figure 1 (step 1). A thin film of 50 nm molybdenum is sputtered at low temperature (50 °C) and etched by dry etching with Cl and O_2_ chemistry. Because of the nature of the sputtering process, Mo covers all the steps in the topography (Supporting Information, Figure S1a). In this way, the CVD-grown graphene will not suffer from any discontinuities. The positive photoresist is then removed by O_2_ plasma, and all the remaining residuals are washed in *N*-methyl-2-pyrrolidone (NMP) with subsequent DI water washing (Figure 1, step 2). At this stage, the graphene is synthesized at 935 °C with an in-house reactor (AIXTRON Black Magic) in 25 mbar of H_2_ as a reducing agent of oxidized Mo and a CH_4_ step for the growth as in Figure 1 (step 3). Next, Cr/Au 20/200 nm is evaporated by ion-beam evaporation in a vacuum and patterned by using a lift-off technique with NMP at 65 °C for 40 min with a final low-power sonication of 90 s (Figure 1, step 4). From NMP the wafer is washed in acetone and DEMI–water. Molybdenum is chemically etched with H_2_O_2_ for 5 min and gently washed with DEMI–water to remove all the chemical residuals (Figure 1, step 5). Deep reactive etching is performed on the backside with the graphene side facing the chuck to avoid any exposure to the SF_6_ plasma that might damage the material (Figure 1, step 6). Finally, after dicing of 1 cm × 1 cm chips, the VHF etch is performed at 45 °C with 100% anhydrous HF, N_2_, and EtOH in a commercially available Primaxx μEtch system at 125 Torr shown in Figure 1 (step 7). Thanks to the high temperature and low pressure, all byproducts related to the undoped SiO_2_ etching are removed by desorption^20^ (Figure S4). No polymers or tapes are involved during the final isotropic etching step since they might trap HF molecules due to their porous nature, and they can usually be only removed by aggressive methods such as O_2_ plasma or other dry-etching chemistry that can damage or remove the suspended graphene. With this proposed approach, a lower temperature of *T* > 110 °C can be used to clean all the residuals that originate from the LPCVD SiN_*x*_ growth and vapor HF reaction,^21^ which is low compared to the *T* > 250 °C cleaning step typically needed to remove the polymer used to transfer the graphene on prepatterned holes. The low temperature in the proposed approach is advantageous because thermal removal of the transfer polymer residuals generally results in a lower number of surviving membranes due to thermal expansion coefficient mismatch of the suspended graphene and the substrate, especially for larger membranes.^9^

### Mechanical Compliance Measurement

The input sound pressure from a speaker is measured with a reference microphone (Sonarworks XREF20) placed next to the sample. A Moku:Lab hardware platform from Liquid Instruments records the signal detected by the reference microphone, the mechanical frequency response of the graphene membrane as detected by using a Polytec vibrometer focused at the center of the membrane, and the output signal of the speaker. After proper correction for the corresponding sensitivities of the vibrometer controller and reference microphone, the mechanical compliance is obtained from the ratio of the two signals received by the Moku:Lab.^22^ Acoustic actuation at a sound pressure level of 1 Pa (≈ 94 dB SPL) is used to test the fabricated membranes.

## Results and Discussion

### Membrane Fabrication Results

**Figure 2:**
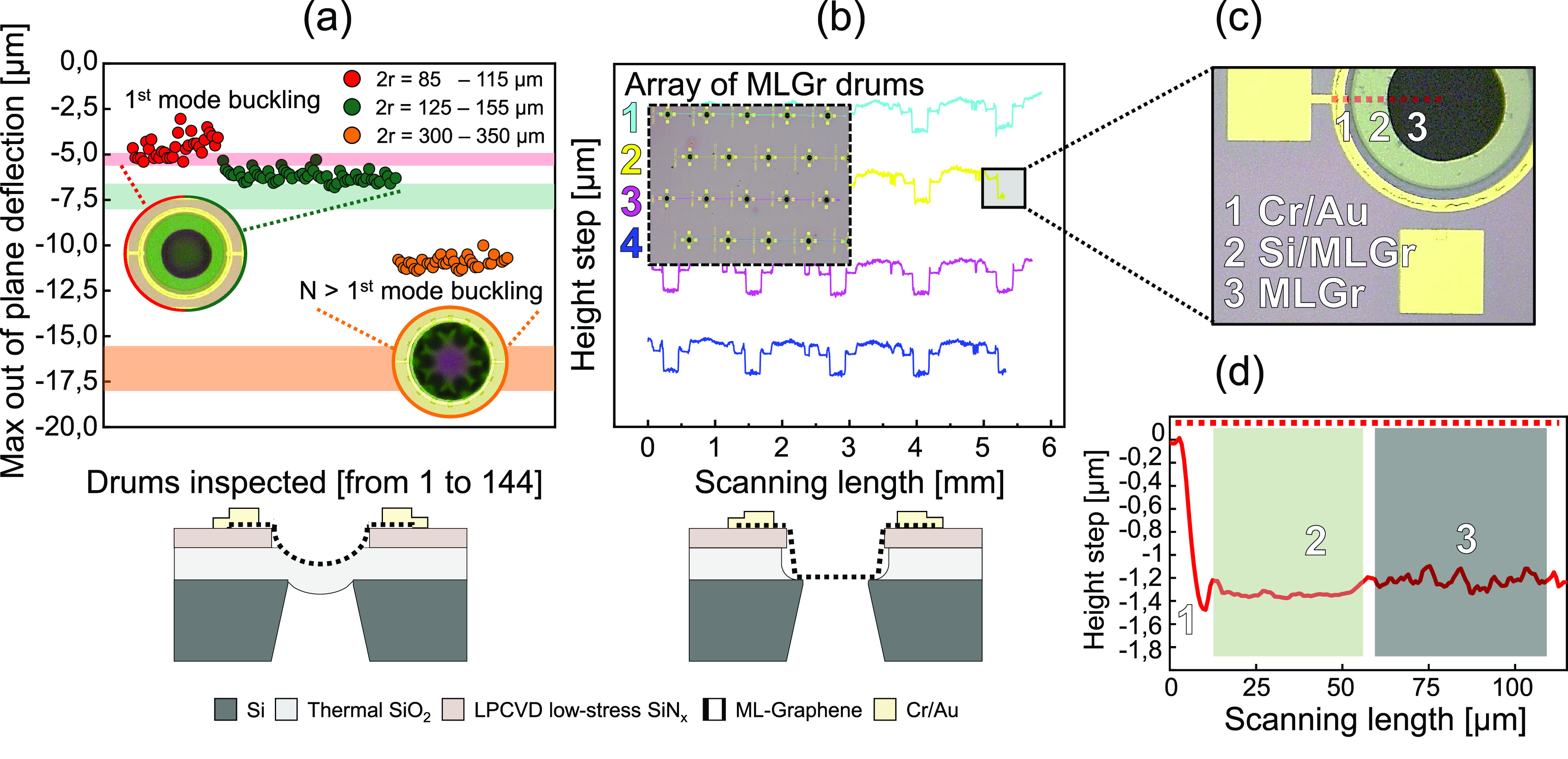
Topographic analysis of the graphene membranes before and after SiO_2_ etch by using 3D laser scanning confocal microscope. (a) Maximum out-of-plane deflection *h*_0_ of the suspended SiO_2_/MLGr heterostructure at the center of the drum. The analytically predicted *h*_0_ for the different diameters, based on a compressive stress in the SiO_2_ of σ = −275 MPa, is indicated by the colored bands. The membranes with 2*r* < 155 μm show an experimental behavior close to the analytical trend with a first buckling mode (inset microscope image *n* = 1). Larger membranes deviate from this trend and reveal higher order modes of buckling (*n* > 1 inset microscope image). (b) Cross section of the wafer containing multiple membranes performed on the released MLGr membranes after the vapor HF removal of the SiO_2_ layer. The membrane on the wafer have reproducible shapes and buckling observed in (a) has disappeared with a flat suspended membrane region. (c) Microscope image showing a Cr/Au electrode for contacting the graphene (not used in this work) and the supported and suspended part of the MLGr. (d) Step height measurement with the confocal microscope along the red dashed line in (c) showing that the MLGr is around 1.3 μm lower than the electrode. This height difference is due to the thickness of the SiO_2_, SiN_*x*_, and electrodes.

The topography of the released SiO_2_/MLGr heterostructure (before HF etch of the SiO_2_) is optically inspected by a 3D laser scanning confocal microscope on more than 100 drums. The graphene is observed to display out-of-plane deformation (Figure 2a) due to compressive stress in the SiO_2_ that causes the diaphragm to bend downward. This unwanted behavior originates mainly from the difference in the thermal expansion coefficients between the SiO_2_ layer and the silicon substrate.^23−25^ For small diameter membranes the first buckling state of the drums is observed (inset Figure 2a), whereas for drums with larger diameters 2*r* = 300–350 μm wavy deformations along the edge of the membrane correspond to higher buckling modes.^26^ In Figure 2a, the maximum values of the out-of-plane deflection *h*_0_ of the center of the membranes are plotted, as determined for different membrane diameters. The deflection of the heterostructures can be modeled by the following analytical equation.^23−25^
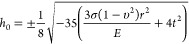
1For the SiO_2_ layer, the Poisson’s ratio υ = 0.2, the Young’s modulus *E* = 74 GPa, the thickness *t* = 0.95 μm, and the radii *r* are reported in the legend of Figure 2a. With these input values, a compressive stress σ in the SiO_2_ layer σ = −275 MPa^24^ is fitted to obtain the observed correspondence that is indicated by the three colored bands in Figure 2a. This analytical calculation is based on the assumption that only the stress σ in SiO_2_ is considered^25^ due to its considerable thickness compared to the other materials involved.

Whereas this model captures the deflection of the small membranes quite well, the deflection *h*_0_ measured on experimental the large diameter drums (2*r* = 300–350 μm) is relatively far from theory, which is attributed to the presence of higher buckling modes.^26^ Once the SiO_2_ is etched, the graphene restores its original flat shape in the suspended region by adhesion to the unetched silicon substrate along its circumference (Figure 2b–d and Figure S1b). We verify its flatness by measuring the step height between the surface of the Cr/Au electrode and the Si surface of the suspended MLGr (Figure 2d), which is ≈1.3 μm, equal to sum of the thickness of the SiO_2_, SiN_*x*_ and the electrode. As can be seen in Figure 2b, the membranes survive the processing. We quantify the yield using optical inspection in Table 1, where membranes with diameters of 85–155 μm show a 100% yield with all 132 suspended devices surviving after the vapor HF release. Large SiO_2_/MLGr heterostructures with diameters of 300–350 μm show a 37% yield on 117 fabricated drums after the DRIE. After SiO_2_ etching, the same drums decrease their yield from 37% to 18%. After 1 week of storage and handling, the small membranes still show 100% of yield, and the survival rate of large membranes decreased to 8%. The difference in yield for the studied drum sizes can be attributed to the high deformations that cause wrinkles, distortions, and even cracks in the oxide (Figure S2). They may negatively affect the graphene integrity creating randomly distributed tears and localized stress. During the vapor HF, handling, and storing they might be a source of crack propagations leading to final membrane breakage. Gas pressure on the membrane during handling and storage might also play a role and account for the yield reduction in the larger membranes. This yield reduction might be mitigated in future processes by tuning the stress and thickness of the oxide layer or by using strain compensation measures like suspending the MLGr from a SiN_*x*_ frame that generates tensile stress.^27^

**Table 1:** Fabrication Membrane Yield (Surviving Membranes/Total Membranes)

diameter	85–155 μm	300–350 μm
MLGr/SiO_2_ at DRIE^a^	132/132	44/117
MLGr at VHF^a^	132/132	22/117
MLGr at 7 days^a^	132/132	10/117

a Survived at the mentioned step without ruptures or cracks.

### Graphene Characterization

**Figure 3:**
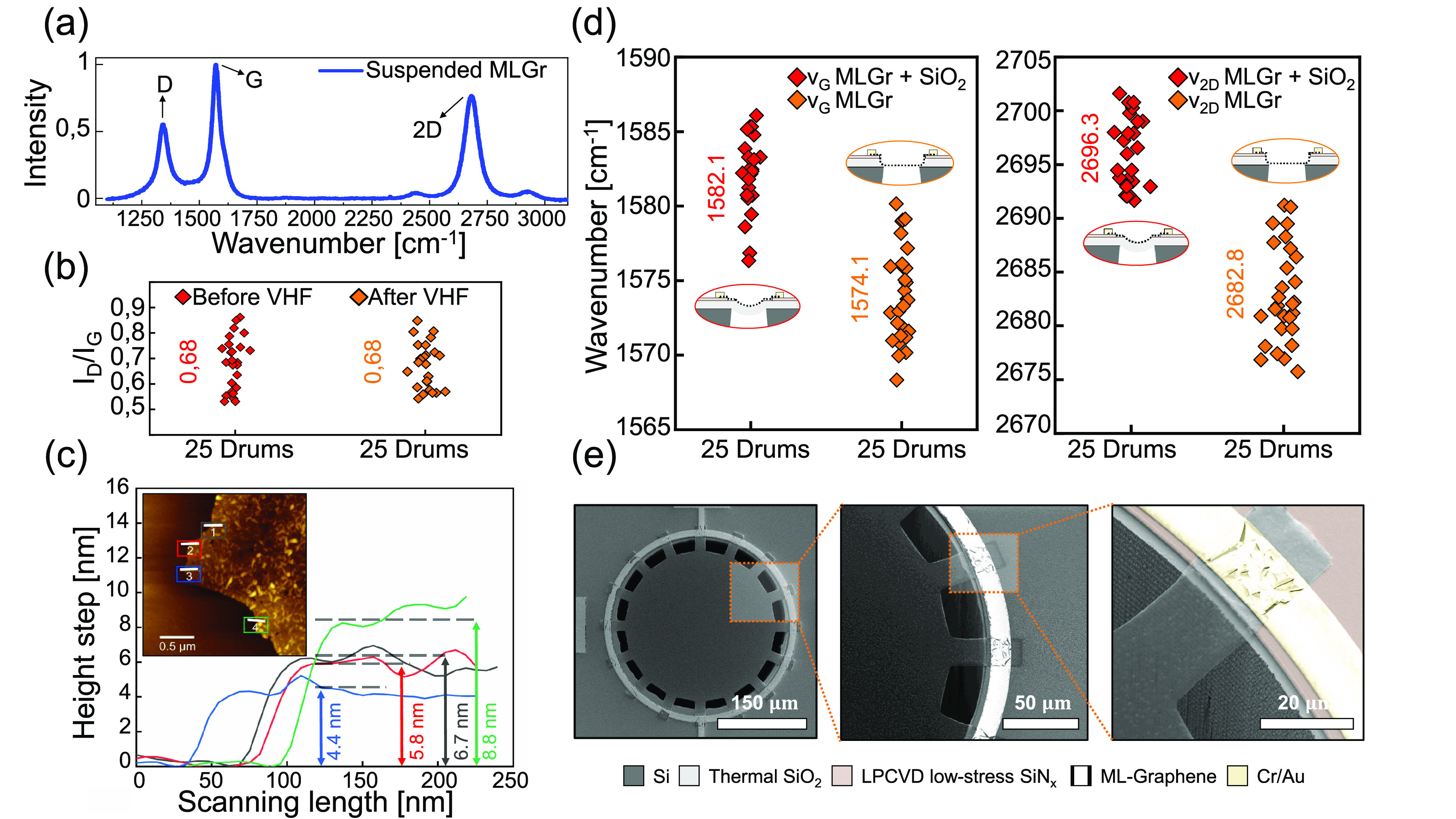
Graphene characterization. (a) Raman spectrum from 1100 to 3200 cm^–1^ of the trampoline MLGr in the suspended region after the VHF release. (b) Comparison of the Raman peak ratio *I*_D_/*I*_G_ before and after the VHF treatment in the suspended region for 25 drums. (c) Thickness measurement of the MLGr graphene after transfer by AFM in semicontact mode. (d) Raman peak frequencies of the G and 2D bands before and after VHF etch in the free-suspended area. (e) Scanning electron microscope pictures made by a SEM Hitachi Regulus 8230 of suspended graphene trampoline with a diameter of 2*r* = 300 μm. Cr/Au electrodes and SiN_*x*_ substrate clamp the suspended graphene membrane.

The crystallinity of the MLGr is investigated through a Horiba HR800 Raman spectrometer equipped with a 514.5 nm Ar^+^ laser maintained at 5 mW to minimize any possible damages by laser heating. A 100× objective with a numerical aperture of 0.9 is used, giving a spot size of about 696 nm. The graphene properties are mainly characterized by two Raman bands: The first is the G band, which is characteristic of all the graphitic sp^2^-type structures and typically centers at 1580 cm^–1^ when the material is in a stress and doping-free state. The second band is the 2D band, which is centered at 2700 cm^–1^ and mainly gives information about the number and stacking order of the layers. In Figure 3a, which shows an example of the Raman spectrum of a MLGr trampoline after VHF normalized to the G band, a third prominent D band is observed that has an intensity of ≈0.68 presenting evidence for the presence of defects. In fact, the D band is related to any kind of defect that distorts the graphene lattice, like edges, wrinkles, Stone–Wales defects, and vacancies. The relatively large D band intensity shows the invasiveness of the process on the graphene compared to previous works where lower defect intensity was reported for the same material.^16,17^ This increased defectivity source is mainly attributed to the lift-off step where *N*-methylpyrrolidone (NMP) is used for the Cr/Au electrode fabrication. The graphene on Mo is exposed for 40 min at 65 °C with a final low-power ultrasonic bath of 90 s to strip the cross-linked photoresist (PR). Intercalation of NMP into the stacked layers and the short ultrasonic bath have probably negatively affected the quality of the material, as already reported in other works.^28−30^ We exclude that the DRIE is invasive since the graphene is not exposed to the plasma etch from the backside of the wafer and instead faces the tool’s chuck. VHF is also not considered a possible defective source because the *I*_D_/*I*_G_ ratio does not show any significant change after the release (Figure 3b). Upon comparison of the G and 2D peak positions of the material before and after the suspension, a prominent red-shift or softening is reported. Shifts of ν_G_ from 1582.1 to 1574.1 cm^–1^ and ν_2D_ from 2696.3 to 2682.8 cm^–1^ are found after the VHF removal of the SiO_2_ from the suspended MLGr (Figure 3d). These shifts might be attributed to phonon softening due to the graphene stress going from a compressive to a more tensile state during the removal of the buckled SiO_2_ layer as illustrated schematically in Figure 2a,b. These results align with previous works reported in the literature on monolayer, few-layer graphene, and graphite.^31−33^ Finally, it can be seen that the Raman 2D-peak ratios are representative for multilayer graphene,^34^ as shown in Figure 3a where *I*_2D_/*I*_G_ < 1 ratios are measured. An atomic force microscope (AFM) from Cypher Asylum Research is used in semicontact mode for the graphene thickness measurement, and a value of ≈7 ± 2 nm is found, where the standard deviation of 2 nm represents its nonuniformity as shown by the variations in the line scans in Figure 3c. Because it is not feasible to directly measure the step height of the carbon layers on the SiN_*x*_, the reported AFM thickness measurements are obtained on graphene which is processed with all the reported steps except the VHF and then wet-transferred in DI water on a clean thermally oxidized silicon chip. The graphene needs to be transferred because SiN_*x*_ is partially etched during the Mo patterning and the VHF exposure and can therefore not be regarded as a flat reference point. The small wrinkles observed in Figure 3c are generally attributed to the small grains of the Mo catalyst surface that arise during the CVD synthesis temperature of 935 °C, whose topography is imprinted into the graphene.^35^Figure 3e shows a suspended membrane that is patterned in a trampoline geometry with a diameter of 300 μm. At the supports of the MLGr trampoline it is evident that the Cr–Au/SiN_*x*_/Si interface acts as a clamping support for the suspended graphene.

### Resonance Frequency and Mechanical Compliance

**Figure 4:**
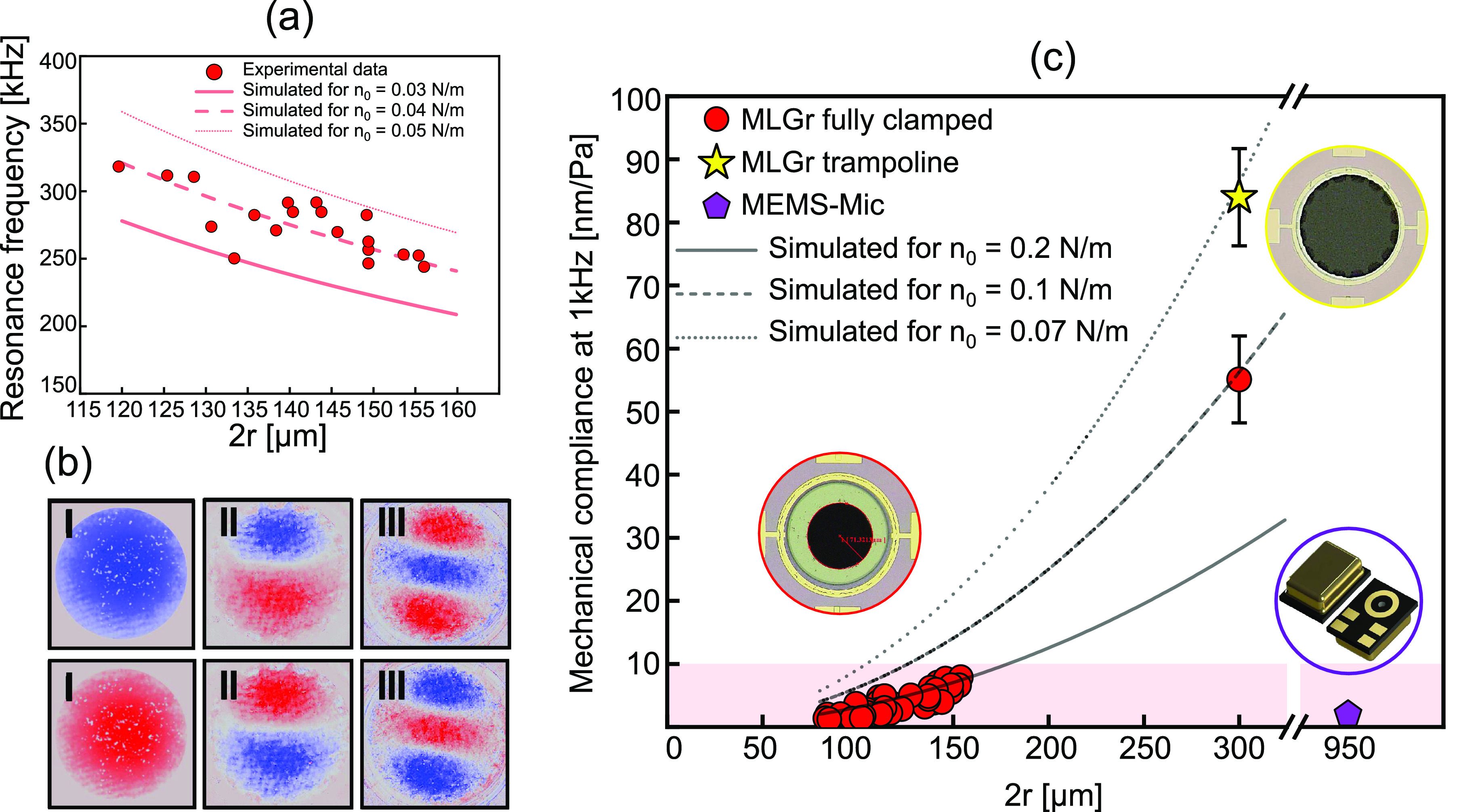
Resonance frequency and mechanical compliance measurements. (a) Resonance frequency as a function of diameter for MLGr membranes with 120–155 um diameters inspected by a LynceeTec holographic microscope. Comparison to eq 2 suggests the tension in the membranes is *n*_0_ = 0.04 N/m. (b) Three first modes of the trampoline membranes recorded by a LynceeTec microscope at resonance frequencies of 92, 136, and 195 kHz. Blue and red colors indicate the phase of the motion, corresponding to upward and downward moving parts of the membrane. The color saturation is a measure for the motion amplitude. (c) Comparison of the mechanical compliance of the MLGr membranes with a commercial MEMS microphone MP23DB01HP, MP34DT04 STMicroelectronics (purple pentagon). MLGr shows comparable and higher mechanical compliance despite its smaller dimension. Large membranes are more sensitive due to their larger diameter. The experimental measurements are also compared with the ideal analytical trend of eq 3 with different pretension values, suggesting that the pretension reduces for larger membranes.

The first mode of the resonance frequency is measured by a digital holographic microscope (LynceeTec) using a laser with a wavelength of 666 nm and a 10× objective lens. The interference between the reflected laser beam from the sample and the reference path provides the intensity and phase of each pixel defining the 3D topography. The measurements are performed in a chamber connected to a vacuum pump at 10^–4^ kPa to reduce air damping effects. The samples are actuated by shaking them with a piezoelectric element below the chip that is actuated by using a voltage-controlled stage with a 0.5 V sine wave in the frequency range *f* = 50–350 kHz where the fundamental resonance frequency is given by eq 2.^36^
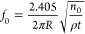
2Here *n*_0_ is the pretension (N/m) of the graphene, ρ the mass density, and *t* the thickness of the graphene. In this relation, the contribution of the bending rigidity is neglected since its is estimated to be small.^37^ The inspected membranes show fundamental frequencies over a range of ≈156–218 kHz for diameters of 2*r* = 120–155 μm (see Figure 4a) and ≈92 kHz for trampolines with diameters of 2*r* = 300 μm. These measured values show lower resonance frequencies compared to previous results obtained for monolayer and bilayer graphene of similar diameter.^9^ Using a graphene thickness of 7 nm, a density of 2260 kg/m^3^, and a diameter range 120–155 μm, the experimental results fit the analytical values in a pretension window of *n*_0_ = 0.03–0.05 N/m (see Figure 4a). The deviations between experimental and theoretical resonance frequencies might be caused by variations in pretension that can be attributed to small nonuniformities in the graphene boundary conditions due to variations in hole geometries from a perfect circular shape in the holes made by using the DRIE etch, differences in the clamping electrode geometries, mass density variations, or unetched SiO_2_ residuals between the graphene and the silicon substrate. When the membrane is actuated at its resonance frequency, its mechanical mode shape is captured by the LynceeTec via the optical phase shifts that result from the oscillating membrane (Figure 4b and Movie S1).

Finally, we characterize the acoustic membrane displacement in the presence of sound at a frequency of 1 kHz at a sound pressure level of 94 dB. The compliance or sensitivity in nm/Pa of the fabricated graphene membranes is determined optically by using a single point laser Doppler vibrometer (LDV) (OFV-5000 Polytec GmbH) at the center of the membrane. Details of the experimental setup and procedure can be found in ref (22) and the Experimental Section. The experimental mechanical compliance at 1 kHz are compared again with the analytical results based on eq 3, which relates the applied pressure to the maximum deflection of the membranes.^3^ Far below the resonance frequency in the linear regime (such that cubic terms in *z* can be neglected), the membrane displacement *z* is related^3^ to the sound pressure level Δ*P* by the equation
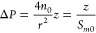
3Thus, a quadratic relation is expected between the mechanical compliance, or sensitivity , and radius *r* for a constant sound pressure level Δ*P*. The reported mechanical compliances of 76 drums shown in Figure 4c range from ≈3–10 nm Pa^–1^ for the smaller membranes with 2*r* = 85–155 μm and ≈43–92 nm Pa^–1^ for large membranes with 2*r* = 300 μm. Figure 4c also shows a MEMS microphone with a membrane diameter of 950 μm, which is measured by using the same procedure resulting in a value of *S*_*m*0_ = 3 nm Pa^–1^.

The graphene data are fit by eq 3, showing that different pretensions *n*_0_ are needed to fit the large (2*r* = 300 μm) and small membranes. Large membranes are closer to the analytical results obtained with lower pretension *n*_0_ = 0.1 N/m, whereas the smaller membranes yield *n*_0_ = 0.2 N/m. This could be attributed to a lower pretension due to the larger suspended region where membrane sagging might be more profound. Moreover, the tension is also affected by the graphene clamping geometry, where for the same diameter of 300 μm the trampoline shows an even higher mechanical compliance (by a factor 1.4) due to lower tension of *n*_0_ = 0.07 N/m compared to the fully clamped geometry.^2^ The mechanical compliance of drums with different sizes and geometries, over the entire audible range, is also reported (Figure S3). When comparing the pretension extracted from eqs 2 and 3, we note that different values of *n*_0_ are obtained as in Figure 4c. These differences might be caused by uncertainty in the mass and thickness that affect eq 2, gas damping and permeation effects at 1 kHz that affect eq 3, and differences in the deflected mode shapes from theory. More study is needed to quantitatively account for these differences. We note that the higher pretensions extracted from eq 3 are the most relevant for device operation as microphone. Furthermore, we demonstrate that music can be recorded by a MLGr drum with 2*r* = 300 μm by monitoring the motion of the graphene membrane using the Polytec vibrometer^22^ (Supporting Information, Audio S1). The output signal from the polytec in response to an arbitrary sound file is recorded with a sampling frequency of 20 kHz to detect the sound waveform, and the measured trace is then reconverted to an audio file with Python.

**Figure 5:**
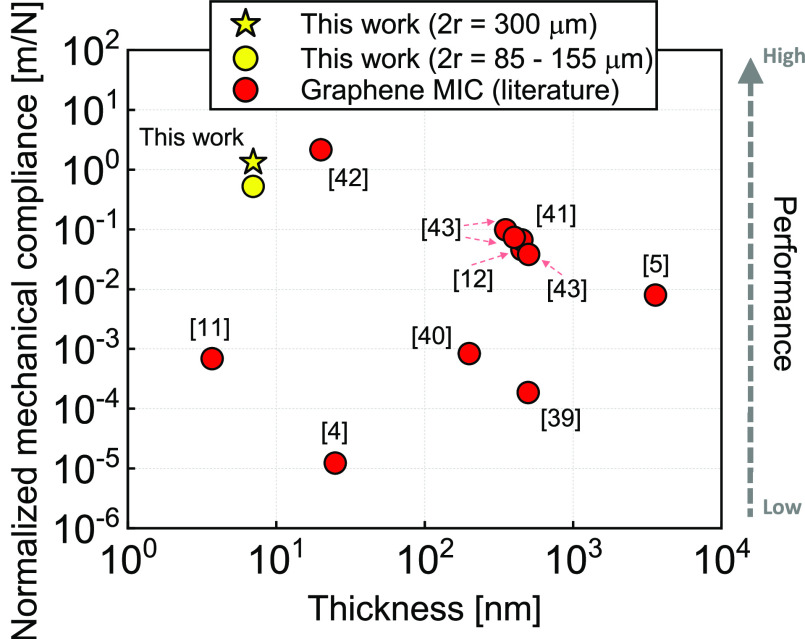
Comparison of the mechanical compliance normalized by the area of graphene microphone. The extrapolated mechanical compliances related to the principal graphene microphone works,^4,5,11,12,39−43^ and the proposed results are presented. The values are normalized by area of the suspended graphene to have an accurate correlation within the different works.

The proposed wafer-scale transfer-free multilayer graphene performances are visually compared in Figure 5 with the transfer-based graphene and graphene heterostructures condenser microphones reported in the literature. It mostly shows higher performance of the presented membranes due to the absence of any polymer supports, leading to higher deflection under sound pressure with also a new fabrication method that is more prone to mass production. Moreover, in these works, the microphone sensitivity *S*, defined as open-circuit sensitivity,^38^ is measured by an electrical read-out. Theoretically, it is equal to the product between the electrical sensitivity *S*_*e*_ and the mechanical sensitivity *S*_*m*0_ contribution by the equation^38^
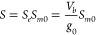
4For this reason, the respective mechanical compliances are indirectly calculated by using eq 4 and eqs 2, 3 as also described by Baglioni et al.^22^ where the input values *S*_*e*_ (m V Pa^–1^ or dB), pretension (N/m), resonance frequency (Hz), the distance membrane–bottom electrode *g*_0_ (m), and *V*_*b*_ (V) are obtained from the reported works.^4,5,11,12,39−43^ For most of the presented data (electrical read-out), the mechanical compliance is indirectly calculated from eq 4. Thus, Figure 5 may show limitations in the comparison with the results obtained by optical read-out (this work). This may be due to the presence of a *V*_*b*_ (bias voltage), resulting in an underestimation of the extrapolated reported mechanical compliance.

## Conclusions

With this work, we present an efficient transferless method to fabricate wafer-scale graphene drums with diameters from 2*r* = 85 to 300 μm. Large arrays of graphene drums with diameters up to ∼155 μm are fabricated with a high yield of 100% by using a CMOS-compatible process flow without any transfer steps. These graphene membranes are shown to operate as microphones, detecting sound with mechanical compliances as high as 92 nm Pa^–1^, which is much higher than that of commercial MEMS microphones that typically have a compliance of 3 nm Pa^–1^. The graphene attains this high sensitivity by using a membrane area that is 10 times smaller than that of the MEMS device. This demonstrates the great potential of graphene for microphone applications. The fabrication route for trampoline designs offers the possibility to invent and engineer new suspended graphene geometries for realizing very high mechanical compliances at the wafer scale. When integrated with electrodes and read-out electronics, it can enable next-generation, high volume, wafer-scale graphene microphone technologies.

## Supporting Information Available

Figure S1: SEM images of the Mo sputtering and graphene conformal coverage at height step; Figure S2: 3D laser confocal microscope images of higher order buckling modes and cracks of SiO_2_/MLGr heterostructures; Figure S3: mechanical compliance of different membranes with 2*r* = 135–155 μm and 2*r* = 300 μm recorded in the audible frequency range; Figure S4: vapor HF and silicon oxide (PDF)
Movie S1: LynceeTec characterization of the motion of a graphene trampoline while driven at its resonance frequency (MOV)
Audio S1: music track (Lorenzo Senni - Canone Infinito) recorded by transfer-free multilayer graphene (ZIP)

## Supplementary Material

am2c03305_si_003.pdf

am2c03305_si_002.mov
