# Supplementary material for: Sensitive Transfer-Free Wafer-Scale Graphene Microphones
Source: ACS Appl Mater Interfaces. 2022 Apr 27;14(18):21705–12. doi: 10.1021/acsami.2c03305 (PMC9100512; doi:10.1021/acsami.2c03305)
Supplement: Supplementary file 1 — am2c03305_si_003.pdf [file am2c03305_si_003.pdf]

Supporting Information: Sensitive Transfer-Free Wafer-Scale Graphene Microphones

Roberto Pezone,[*,†] Gabriele Baglioni,[‡] Pasqualina M. Sarro,[†] Peter G. Steeneken,[¶,‡] and Sten Vollebregt[*,†]

†*Laboratory of Electronic Components, Technology and Materials (ECTM), Department of Microelectronics, Delft University of Technology, The Netherlands*

‡*Kavli Institue of Nanoscience, Department of Quantum Nanoscience, Delft University of Technology, the Netherlands*

¶*Department of Precision and Microsystems Engineering (PME), Delft University of Technology, The Netherlands*

E-mail: r.pezone@tudelft.nl,s.vollebregt@tudelft.nl;

Phone: +31 152789437;

S1. Molybdenum and graphene step coverage

In Figure S1a the Mo/MLGr coverage at the vertical sidewalls interface SiN_x/SiO_2 is shown. The Figure S1b confirms the graphene continuity at the interface SiN_x/SiO_2 after the VHF release. Here, the MLGr is clamped by the SiN_x and silicon substrates.

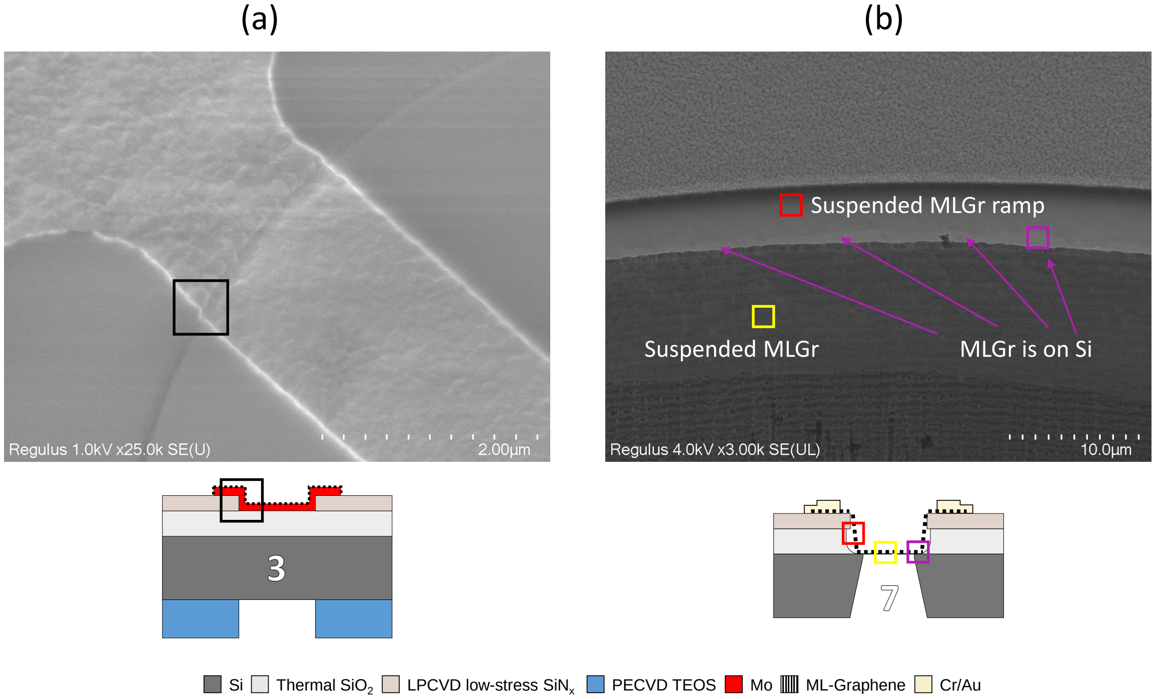

Figure S1: SEM images of the Mo/MLGr at the SiN_x/SiO_2 interface (a) and graphene conformal coverage at height step (b).

S2. Buckling N>1-modes effects

The Figure S2 shows the SiO_2/MLGr deformation after the DRIE of silicon substrate in corrispondence of the opening. Large deformation, wrinkles, cracks lead to low yield of final MLGr membranes with diameter ranging from 300 µm to 350 µm.

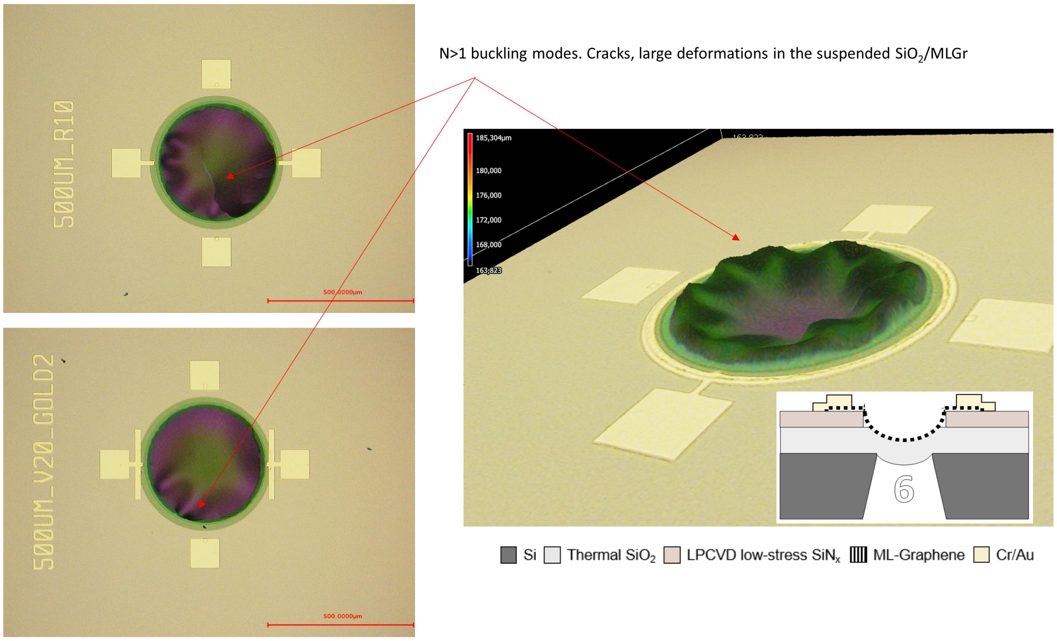

Figure S2: 3D laser confocal microscope images of higher order buckling modes and cracks of SiO_2/MLGr heterostructures.

S3. Mechanical Compliance in the audible range

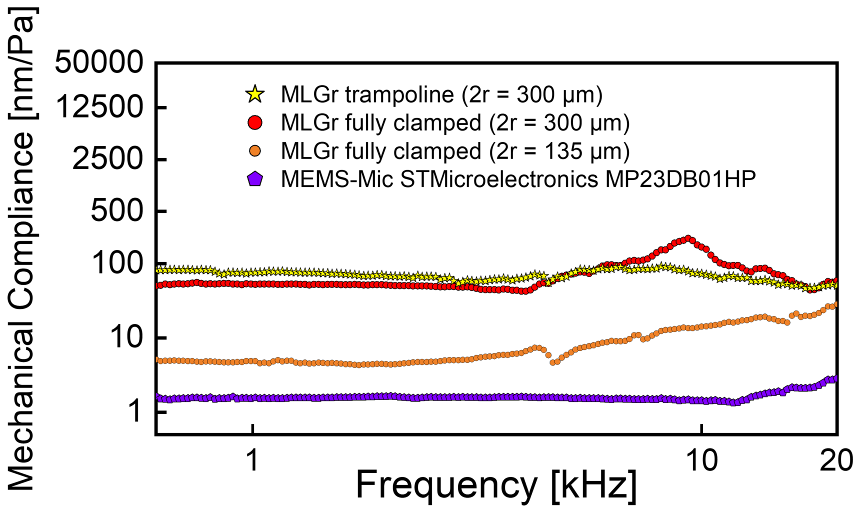

Figure S3: Mechanical compliance of different membranes with $2r = 135\,\mu\text{m}$ and $2r = 300\,\mu\text{m}$ recorded in the audible frequency range.

S4. Vapor HF and Silicon Oxide

Adsorption/Ionization (1)

HF (gas) ↔ HF (ads) ● A (gas) ↔ A (ads)

2HF (ads) + A (ads) → HF_2^- (ads) + AH^+(ads)

Main etch (2)

SiO_2 (s) + 2HF_2^- (ads) + 2 AH^+(ads) → SiF_4 (ads) + 2H_2O (ads) + 2A(ads)

Desorption (3)

SiF_4 (ads) ↔ SiF_4(gas)

H_2O(ads) ↔ H_2O(gas)

A(ads) ↔ A(gas)

Figure S4: Chemical reaction between Vapor HF and silicon oxide.[1]

References

(1) Hammond, P. *Handbook of Silicon Based MEMS Materials and Technologies*; Elsevier, 2015; pp 540–549.
